# Acute Marjolin's Ulcer Arising in a Split-Thickness Skin Graft Postburn Injury

**Published:** 2016-07-25

**Authors:** Philicia Moonsamy, Rosalynn M. Nazarian, John T. Schulz, Jeremy Goverman

**Affiliations:** ^a^Division of Burn Surgery, Department of Surgery, Massachusetts General Hospital, Boston; ^b^Dermatopathology Unit, Pathology Service, Massachusetts General Hospital, Boston

**Keywords:** acute Marjolin's ulcer, burn, skin graft, nonhealing wound, squamous cell carcinoma

## DESCRIPTION

A 44-year-old man sustained a 55% total body surface area burn secondary to a gasoline fire. He required numerous rounds of excision and autografting and was discharged to inpatient rehabilitation on postburn day 45. Approximately 3 months postburn, he presented to clinic with a persistent area of hypertrophic granulation tissue overlying his left tibia at the site of a previous full-thickness skin excision and split-thickness skin graft. The lesion was found to be squamous cell carcinoma (SCC).

## QUESTIONS

1. **What are the predisposing factors and average time at presentation of a Marjolin's ulcer?**
2. **What are the histological findings?**
3. **How are these lesions diagnosed and treated?**
4. **What is the prognosis?**

## DISCUSSION

Marjolin's ulcers are rare cutaneous neoplasms that arise in previously traumatized skin, especially previous burn injuries. Patients present in a bimodal distribution with acute and chronic types. Acute is defined as a malignancy that occurs within 12 months of the original injury and usually manifests as basal cell carcinoma. The shortest time interval reported in the literature between injury and histological confirmation of malignancy is 4 to 6 weeks. The chronic type is much more common overall, as the malignancy tends to develop very slowly, with an average time for malignant transformation of 35 years. The chronic form classically presents as SCC and is thought to arise in deeper, full-thickness burns in contrast to the acute form, which is associated with smaller, partial-thickness burns. The lag period before a malignancy arises is inversely proportional to the patient's age at the time of the initial burn injury and is more common in men than in women.^1^^,^^2^

**Figure 1:**
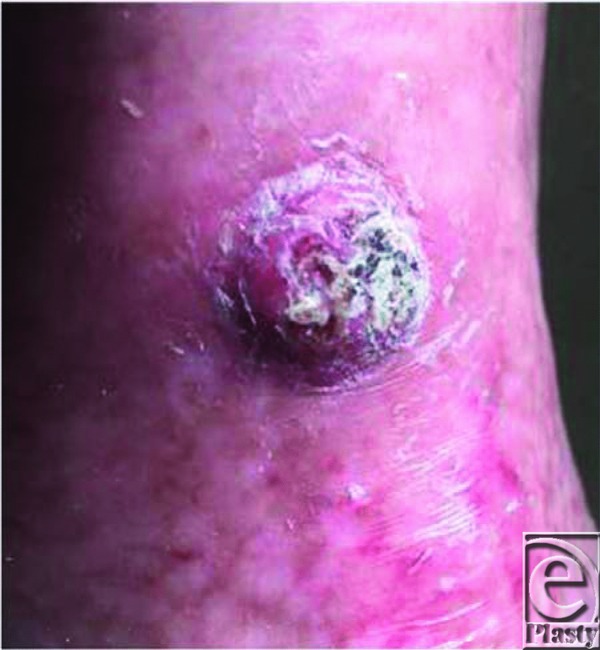
Nonhealing hypertrophic left tibial lesion arising in burned skin that was autografted 3 months prior.

**Figure 2:**
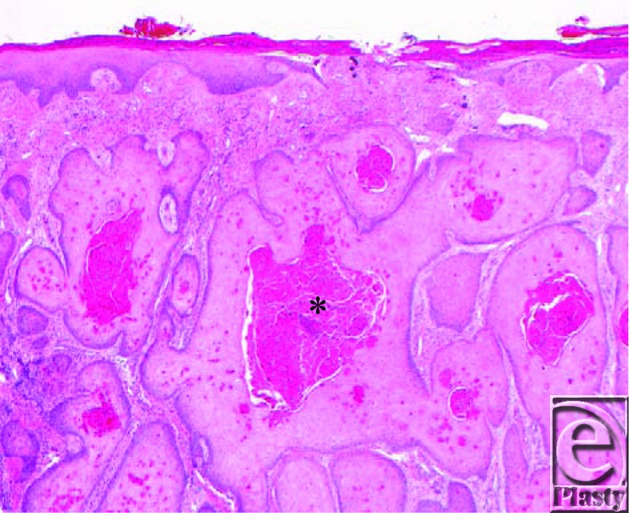
Left tibial skin histopathology demonstrates irregular nests of atypical squamous epithelial cells, with central keratinization (asterisk) extending to the reticular dermis consistent with invasive well-differentiated squamous cell carcinoma (hematoxylin-eosin, original magnification ×40).

**Figure 3:**
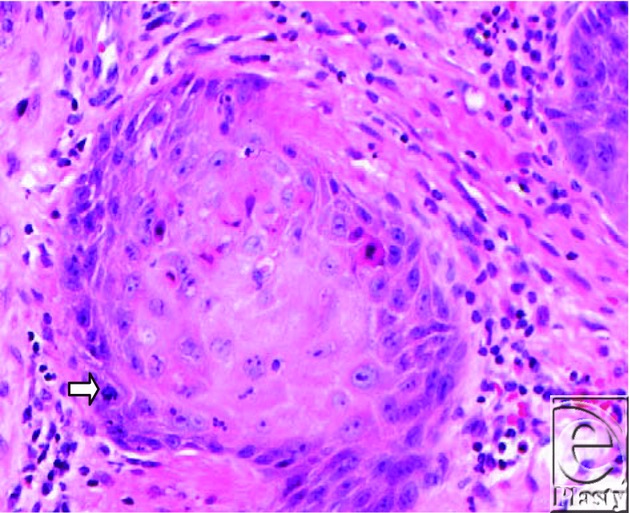
At higher power, nests of pleomorphic enlarged squamous cells with abundant eosinophilic cytoplasm and atypical mitotic figures (arrow) are seen in a background of granulation tissue (hematoxylin-eosin, original magnification ×400).

The term “Marjolin's ulcer” is used collectively to describe SCC, basal cell carcinoma, malignant melanoma, and mesenchymal tumors; however, the most common type of histology seen is well-differentiated SCC. Burn scar carcinomas usually present as flat ulcerated lesions with elevated margins and surrounding induration. They less commonly present as exophytic lesions that resemble granulation tissue, as in this patient (Fig 1). Histopathological evaluation of his left tibial skin lesion revealed an atypical squamous proliferation characterized by irregular nests of keratinizing squamous epithelium extending to the deep dermis consistent with well-differentiated invasive SCC (Fig 2). The tumor was present in a background of granulation tissue (acute and chronic inflammatory infiltrate, vascularity, and fibrosis) with foci of epidermal ulceration, compatible with Marjolin's ulcer (Fig 3).

**Figure 4:**
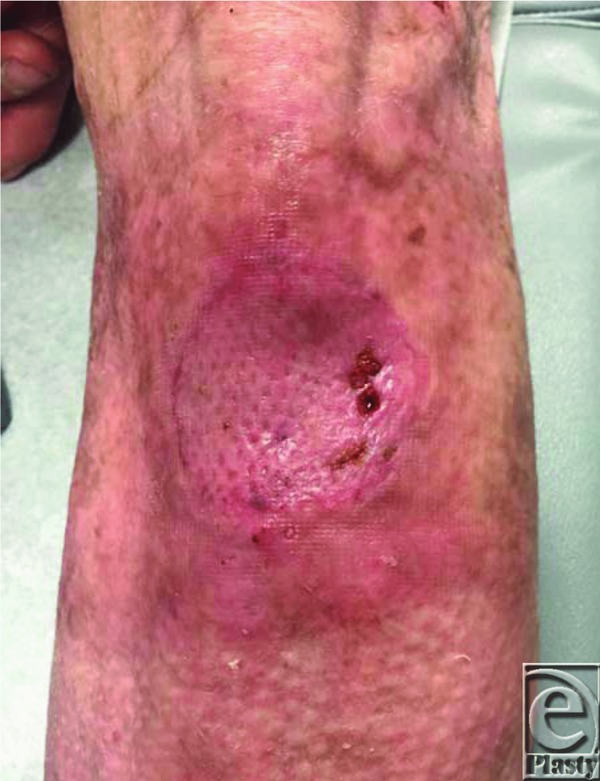
Left shin after excision of lesion and autografting with split-thickness skin graft.

The diagnosis is made by taking a thorough history, which typically includes a chronic nonhealing wound/scar. Biopsy is indicated if there is suspicion of malignancy, and lymph node examination should be performed on all patients. Some studies suggest using ultrasound to assess lymph nodes; however, there is no current consensus on the indications for lymph node dissection. Radical excision is the current treatment of choice, with 2-cm surgical margins (Fig 4).

Marjolin's ulcers are considered to be more aggressive than conventional SCC of the skin, and they have a higher potential for early metastasis. The rate of SCC metastasis currently reported in the literature is 0.5% to 3.0%, which is in contrast to a metastasis rate of 30% to 34% in SCC originating from a burn scar. There have not yet been any studies to establish clinical or prognostic differences between the acute and chronic forms.^1^^,^^2^
